# Mechanisms of Nanotoxicology and the Important Role of Alternative Testing Strategies

**DOI:** 10.3390/ijms23158204

**Published:** 2022-07-26

**Authors:** Yuan-Hua Wu, Sheng-Yow Ho, Bour-Jr Wang, Ying-Jan Wang

**Affiliations:** 1 Department of Radiation Oncology, National Cheng Kung University Hospital, College of Medicine, National Cheng Kung University, Tainan 704, Taiwan; wuyh@mail.ncku.edu.tw; 2 Department of Radiation Oncology, Chi Mei Medical Center, Tainan 736, Taiwan; shengho@seed.net.tw; 3 Graduate Institute of Medical Sciences, Chang Jung Christian University, Tainan 711, Taiwan; 4 Department of Cosmetic Science and Institute of Cosmetic Science, Chia Nan University of Pharmacy and Science, Tainan 71710, Taiwan; 5 Department of Occupational and Environmental Medicine, National Cheng Kung University Hospital, Tainan 70403, Taiwan; 6 Department of Environmental and Occupational Health, College of Medicine, National Cheng Kung University, Tainan 704, Taiwan; 7 Department of Medical Research, China Medical University Hospital, China Medical University, Taichung 404, Taiwan

Recently, rapid advances in nanotechnology have provided a lot of opportunities for the mass production of engineered nanomaterials of various types of chemicals, including metals and nonmetals, promoting the development of a new generation of industrial and commercial products and the field of nanomedicine. However, the hazardous impacts of nanomaterials on both human beings and the environment have not yet been thoroughly clarified [1]. To evaluate the toxicity of the huge range of nanomaterials available, safety assessments with traditional animal studies need to be conducted. Due to the fact that traditional animal approaches are unethical, expensive, and time-consuming, there is a need for developing alternative testing strategies and methods that reduce, refine, or replace (3Rs) the use of animals for assessing the toxicity of nanomaterials. Understanding the mechanisms of toxicity triggered by nanomaterials is fundamental for the development of alternative testing methods. In addition, the use of common and reasonable biomarkers for toxicity evaluations is also important [2].

Nanomaterials may enter the human body through different routes and be transported into organs with the help of blood and lymph systems. In the application of newly developed nanocarriers in the field of drug delivery, the unwanted accumulation of these substances in organs outside of their targeted site of action may lead to severe toxicological side effects. Previous studies have shown the unintended accumulation of various nanocarriers in female sex organs, especially in the ovaries. A recent report indicated that the ovarian accumulation of nanocarriers such as nanoemulsions depends on both the age of the patients as well as the particle size during maturity [3]. In addition, neuroinflammation and the hormonal control of the hypothalamus via the gonadotropin-releasing hormone receptor exposed to cadmium-selenium quantum dots have been examined. The results of studies indicate that alterations in the hormonal control of the hypothalamus may have a potential adverse effect on fertility [4].

Only a few studies have investigated the effects of nanomaterial toxicity on immunological functions. In research using a zebrafish model, the toxic effects of silver nanoparticles (AgNPs) on innate immunity was examined. The results indicated that AgNPs induced toxic effects in zebrafish, including death; malformation; and innate immune toxicity, such as changes in the number and function of neutrophils and macrophages. Furthermore, the expression of immune-related cytokines and chemokines was also affected [5]. Another study revealed the in vivo effect of aluminum oxide fine particles (Al_2_O_3_ FPs) on the innate and adaptive immune cells in mice. The researchers found that Al_2_O_3_ FPs promoted the activation of spleen macrophages, increasing the number of CD4 and CD8 T cells, which differentiated into interferon-gamma (IFN-γ)-producing helper T1 (Th1) and cytotoxic T1 (Tc1) cells, potentially contributing to the exacerbation of inflammatory diseases [6].

Besides the common application of engineered nanomaterials, plastic particles could also pose an urgent threat to human health, due to the increasing accumulation of plastic waste and its derivatives, micro- and nanoplastics (MNPLs). MNPLs induce several cell responses and are engulfed by cells depending on their size, which in turn triggers autophagy in various cells and tissues [7]. Regarding the responses of cells exposed to MNPLs, it has been reported that polystyrene nanoparticles with various diameters lead to increased ROS levels and induce lipid and protein oxidation, before finally decreasing the viability of peripheral blood mononuclear cells (PBMCs) [8]. Particles with a smaller diameter exhibited a stronger oxidative potential in PBMCs. Interestingly, MNPLs could coexist with other known hazardous contaminants such as arsenic in the environment, thus modulating their uptake and harmful effects. An in vitro cell transformation assay was conducted to evaluate the long-term co-exposure effect of nanoplastics and arsenic. The results indicated that continuous co-exposure enhances DNA damage and the aggressive features of the initially transformed phenotype. Several oncogenic biomarkers have been observed, including a higher proportion of spindle-like cells, an increased capacity to grow independently of anchorage, as well as enhanced migrating and invading potential [9].

Regarding the biomarker of nanotoxicity, one study investigated whether surfactant protein D (SP-D) could serve as a biomarker of the pulmonary toxicity of nanomaterials. The intratracheal instillation of high-toxicity (nickel oxide and cerium dioxide) and low-toxicity (titanium dioxides and zinc oxide) nanomaterials was conducted and rat biological samples (blood and BALF) taken from 3 days to 6 months after installation were collected. The results indicated that the expression of SP-D in serum and BALF depended on the level of lung inflammation and that SP-D can serve as a biomarker for evaluating the pulmonary toxicity of nanomaterials [10]. Another study investigated the impact of copper-titanium dioxide nanocomposite (Cu/TiO_2_-NC) on bacteria viability, antioxidant enzymes, and fatty acid profiling. Microscopic images indicated the occurrence of distinct interactions of the Cu/TiO_2_-NC with the bacterial outer layers. In addition, the biocidal effects of Cu/TiO_2_-NC can be attributed to the induction of oxidative stress, the release of metal ions, and specific electrochemical interactions with the bacterial cells [11].

Currently, the 3Rs principle (replacement, reduction, and refinement) is applied to ensure the more ethical application of animals in studies. Thus, the use of alternative strategies to animal testing has been encouraged to overcome the drawbacks of animal experiments. The available alternative methods include in vitro and in silico approaches, which are regarded as non-animal approaches and have been implemented in many countries for scientific purposes. In vitro experiments related to nanotoxicity involve cell culture testing and tissue engineering, while in silico methods include prediction using molecular docking, molecular dynamics simulations, and quantitative structure–activity relationship (QSAR) modeling [12]. These cell-based methods and computational approaches have the potential to minimize the use of animals for the assessment of nanomaterial toxicity. In silico modeling that combines experimental and computational approaches presents another option to predict the human health risks of nanomaterials and provides a powerful basis for understanding biological mechanisms at molecular levels [13]. For example, machine learning techniques have been applied in neurotoxicity classification models for diverse nanoparticles with encouraging results. A classification model was developed using random forest and goodness of fit with additional robustness and predictability metrics were used to evaluate its performance. The five most important attributes for predicting neurotoxicity in vitro are exposure dose and duration, toxicological assays, cell type, and zeta potential. The model was found to perform better than non-tissue specific models [14]. In light of this, the focus of nanotoxicology research has shifted from traditional animal tests to developing novel in vitro, ex vivo, in silico, and combined strategies for mechanistic exploration.

Recent experimental toxicology approaches are being promoted rapidly by the incorporation of novel techniques that provide a more in-depth view into the mechanisms of the adverse effects of chemical exposure [2]. Assessments of the potential hazards associated with nanotechnology have been emerging, but substantial challenges still need to be conquered. A thorough understanding of the mechanisms by which nanomaterials perturb biological systems is critical for a more comprehensive elucidation of nanotoxicity.
